# Cross-Talk between Lipoproteins and Inflammation: The Role of Microvesicles

**DOI:** 10.3390/jcm8122059

**Published:** 2019-11-22

**Authors:** Gemma Chiva-Blanch, Lina Badimon

**Affiliations:** 1 Cardiovascular Program ICCC, Institut de Recerca Hospital Santa Creu i Sant Pau—IIB Sant Pau, Sant Antoni Maria Claret, 167, 08025 Barcelona, Spain; gchiva@santpau.cat; 2 CIBER Enfermedades Cardiovasculares (CIBERCV), Instituto de Salud Carlos III (ISCIII), 28029 Madrid, Spain

**Keywords:** microvesicles, inflammation, lipoproteins, LDL cholesterol, microparticles, cardiovascular disease, platelets, endothelial cells, leukocytes, atherothrombosis

## Abstract

Atherothrombosis is the principal underlying cause of cardiovascular disease (CVD). Microvesicles (MV) are small blebs originated by an outward budding at the cell plasma membranes, which are released in normal conditions. However, MV release is increased in pathophysiologic conditions such as CVD. Low density lipoprotein (LDL) and MV contribute to atherothrombosis onset and progression by promoting inflammation and leukocyte recruitment to injured endothelium, as well as by increasing thrombosis and plaque vulnerability. Moreover, (oxidized)LDL induces MV release and vice-versa, perpetuating endothelium injury leading to CVD progression. Therefore, MV and lipoproteins exhibit common features, which should be considered in the interpretation of their respective roles in the pathophysiology of CVD. Understanding the pathways implicated in this process will aid in developing novel therapeutic approaches against atherothrombosis.

## 1. Introduction

Atherothrombosis is the principal underlying cause of cardiovascular disease (CVD). Atherosclerosis is caused by lipid accumulation associated with endothelial dysfunction and chronic low-grade inflammation and oxidative stress. Innate immunity cells and vascular smooth muscle cells respond to these perturbations by initiating interactions and gene programs that contribute to vascular dysfunction and atherosclerotic plaque formation. Advanced atherosclerotic plaques, with a lipid-rich atheroma, show inward remodeling encroaching in the arterial lumen, which decrease blood flow, thus leading to tissue ischemia. Eventually, atherosclerotic plaques can rupture and provoke thrombus formation that may occlude the lumen interrupting oxygen supply [1].

High plasma concentrations of low density lipoprotein (LDL) cholesterol induce atherosclerosis, while decreasing LDL cholesterol levels associates with a reduced incidence of major CV events [2]. In fact, the life-long exposure of an artery to LDL cholesterol remains a principal determinant of atherosclerotic progression [3]. Recently, enhanced perivascular adipose tissue mass and local inflammation has been associated with increased atherosclerotic plaque burden [4], and immunity cells and inflammation have a causal role in atherosclerosis progression by modulating the resident cells in the artery wall [3]. 

Microvesicles (MV) derived from blood and vascular cells seem also being able to participate in the initiation, progression and complications of atherothrombosis by a direct and a paracrine regulation of target cells. Therefore, this review is aimed at summarizing the crosstalk between lipoproteins, inflammation, and microvesicles in the pathophysiology of atherothrombosis.

## 2. Atherosclerosis: Lipids and Inflammation 

Atherosclerosis is considered a lipid-initiated, chronic and progressive inflammatory systemic disease of large and medium arteries and characterized by inflammatory and immune responses contributing to the onset and evolution of the disease and to plaque instability and rupture. It is triggered by the presence of elevated levels of cholesterol in the vessel wall. On the other hand, high density lipoprotein (HDL) particles, when functionally capable, can exert the opposite effect by removing cholesterol from the circulation through the induction of reverse cholesterol transport, by protecting LDL and other proteins from oxidative damage [5], and by inhibiting monocyte production of inflammatory cytokines [6,7] and monocyte differentiation [8]. However, alterations in the HDL structure and function can render dysfunctional HDL particles that may exert deleterious effects in the CV system [9,10]. Inflammation plays a pivotal role in atherosclerotic plaque formation and instability by promoting endothelial cell activation, endothelial dysfunction, loss of integrity and lipid deposition in the intima and by impairing endothelial-repairing capacity [11]. 

The involvement of inflammation besides LDL in atherosclerosis has been recently proven in the proof-of-concept CANTOS (Canakinumab Anti-Inflammatory Thrombosis Outcome Study) trial, showing that interleukin 1β inhibition resulted in a 15% reduction in cardiovascular events [12]. Supporting these findings, it has been recently observed that interleukin 6 trans-signaling increases the risk of cardiovascular events [13]. Interleukin 1β/ interleukin 6 signaling is activated by the nucleotide-binding leucine-rich repeat-containing pyrine receptor 3 (NLRP3) inflammasome platform [14]. However, (minimally modified) LDL activates the inflammasome [15,16,17], in its turn activating the Interleukin 1β/ interleukin 6 signaling pathway, and thus hampering the dissection of the separate roles of lipids and inflammation in atherosclerosis progression.

Atherosclerosis initiates with increases in endothelial permeability to circulating LDL and its accumulation in the intima. Activated endothelial cells then release cytokines, chemokines, and adhesion molecules attracting circulating leukocytes, and more specifically monocytes, into the atherosclerotic lesion, inducing the differentiation of monocytes into proinflammatory macrophages and finally foam cells [18]. Although the primary origin of foam cells in atherosclerotic lesions are leukocytes [19], smooth muscle cells have been shown to differentiate to foam cells [20]. The rupture of the atherosclerotic plaque is the most common trigger of thrombosis, leading to an infarction.

Therefore, atherosclerosis is driven by LDL and inflammation mediated by immune cells, leading to thrombosis and a major CV event. In this process, MV act as a paracrine complementary system ensuing atherothrombosis as will be further discussed.

## 3. Microvesicles

MV belong to the family of extracellular vesicles. Extracellular vesicles are literally defined by the International Society for Extracellular Vesicles as “the generic term for particles naturally released from the cell that are delimited by a lipid bilayer and cannot replicate, i.e. do not contain a functional nucleus” [21]. MV are medium/large extracellular vesicles (0.1–1 µm) originated by an outward budding at the plasma membrane from almost all cell types [21,22,23,24], and their release is increased when activated, injured or undergoing apoptosis. The biogenesis of MV requires a structured rearrangement of the plasma membrane which provokes physical bending of the membrane and restructuring of the underlying actin cytoskeleton, inducing membrane budding, exposure of phosphatidylserine (PS) from the inner leaflet to the cell surface and formation of membrane blebs. However, MV biogenesis also occurs even if membrane lipid asymmetry is maintained [25]. Given their biogenesis, they contain bioactive molecules from their parental cells, and lipids are essential components of MV. Cholesterol is a structural component of the plasma membrane, which regulates cell functionality and subcellular compartmentalization, and is a precursor or cofactor for several signaling molecules/pathways. In fact, MV are enriched in cholesterol compared to plasma membranes [26,27], and cholesterol mediates their formation and release, ensures membrane stability, and it is necessary for their uptake by target cells. Although MV are known to carry biomolecules such as proteins, RNAs and even DNA, their complete molecular fingerprint is largely unexplored [28], probably because the molecular composition of MV is largely influenced by the stimulus originating them [29,30]. 

Once released from the cell, MV reach target cells and transfer their molecular cargo, triggering functional responses and phenotypic changes affecting cell functionality, both at the local and systemic environments. The net effect of MV in target cells involves signaling at multiple levels including their interaction with cell surface receptors, and transference of genetic material, metabolites and cytosolic enzymes.

Although the current review is focused in the lipid-mediated role of MV in the pathogenesis of atherothrombosis, it is worth mentioning that there are smaller extracellular vesicles sized ≤150 nm called exosomes, formed by the fusion of intracellular multivesicular bodies with the plasma membrane [23]. Exosomes are rich in biomolecules such as protein, mRNA and non-coding RNA such as miRNA, and have also emerged as both regulators and biomarkers of CVD progression [31]. For further information please refer to reviews [32,33,34,35,36,37]. 

## 4. Role of Microvesicles in Atherosclerosis

As previously mentioned, MV are released from all cell types, including platelets, endothelial cells, smooth muscle cells, leukocytes and erythrocytes. Therefore, MV are released in the bloodstream affecting local and distal vulnerable zones. 

MV are involved in CVD progression by supporting cellular cross-talk leading to vascular inflammation and dysfunction, leukocyte adhesion and tissue remodeling, thus creating an inflammatory and prothrombotic milieu. In parallel to LDL, MV accumulate and promote the progression of atherosclerotic plaques [38]. Given the externalization of PS during their biogenesis, and, in some cases, tissue factor on their surfaces, MV are potent inducers of coagulation [39], and within the atherosclerotic plaque, retained MV account for the procoagulant activity of the lipid core. MV are the main reservoir of tissue factor activity, thus MV increase plaque vulnerability by promoting coagulation after erosion or rupture of the atherosclerotic plaque. In fact, MV exposing PS in their surface enhance clot propagation, cause apoptosis of nearby cells, and modulate gene expression via a variety of pathways [25], for instance, by transferring monomeric C-reactive protein (CRP) to the cell surface and generating pro-inflammatory signals [40]. Endothelial release of MV is associated to increased cytokine production, and in its turn, increased cytokine production increases MV release. In fact, it has been observed that pharmacological attenuation of inflammation induces a decreased shedding of endothelial-derived MV [41]. Monocyte-derived MV increase endothelial thrombogenicity and apoptosis in vitro by increasing endothelial tissue factor expression, also by the adherence of tissue factor derived from MV to the endothelial cell membrane, by decreasing levels of tissue factor pathway inhibitor (TFPI) and thrombomodulin, and by disrupting endothelial integrity [42]. In addition, MV can transfer cluster of differentiation 40 ligand (CD40L) which binds to monocyte CD40 triggering the activation of tumor necrosis factor (TNF) receptor-associated factor 6 (TRAF6) downstream signaling, activating in its turn nuclear factor (NF)-kB, thus inducing monocyte release of inflammatory mediators [43]. Besides their prothrombotic activity [44], platelet-derived MV enhance the expression of adhesion molecules and trigger the production of interleukins and TNF-α [45]. In addition, platelet MV recruit activated platelets to the endothelial injury area and can also activate platelets; and platelet-derived MV also contribute to atherogeneis by inducing smooth muscle cell proliferation [46]. Finally, it has been shown that erythrocytes can induce the vulnerability and rupture of plaques in a dose-dependant manner [47]. This effect has been proposed to be indirectly mediated by erythrocyte MV by activating the endothelium, attracting leukocytes and platelets, and enhancing the whole inflammatory pathway [46], although scarce research has been done in this direction.

Therefore, MV contribute to atherothrombosis progression by indirectly elevating the thrombotic risk through the induction of an inflammatory response and directly activating platelets and coagulation. Although up to 200-fold elevated concentrations of MV have been found at the local area of atherosclerotic plaques compared to plasma circulating levels [48], circulating MV from CVD patients [49] and healthy subjects [44] have shown significant prothrombotic activity per se, a role that should not be underestimated in atherothrombotic progression.

### Crosstalk between Lipoproteins and MV

**Figure 1:**
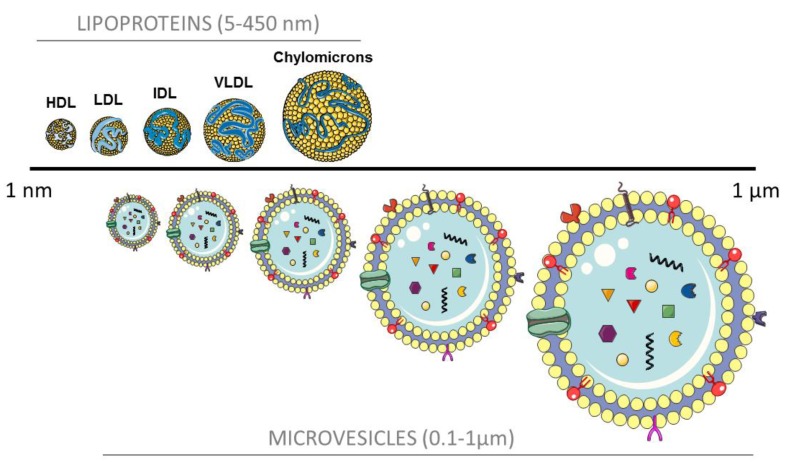
Particle size of lipoproteins and microvesicles. HDL, high density lipoprotein; LDL, low density lipoprotein; IDL, intermediate density lipoprotein; VLDL, very low density lipoprotein; and MV, microvesicles.

Recent findings pinpoint some similarities between MV and lipoproteins: both MV and HDL and LDL contain and transfer miRNA to target cells [50,51]; MV extracts have been shown to carry ApoE, ApoB, ApoC-II, among others; and the size and density of small- and middle-sized MV closely overlap with lipoproteins, as can be observed in Figure 1. As a matter of fact, some isolation techniques result in co-purification of MV and lipoprotein particles [22,23,52]. 

Given this size overlap, it is of extreme importance that blood extraction for MV isolation and characterization is being performed in fasting conditions. Depletion of lipoproteins for MV analyses and vice-versa should be carefully considered in biomarker and functional studies. However, the half-life of lipoproteins and MV differs significantly. In physiological conditions, remnant chylomicrons, LDL and HDL, are readily metabolized and stored as intracellular lipid droplets in the liver and adipose tissue. Oppositely, MV rapidly accumulate in resident macrophages of the liver, lungs, and spleen [53]. During atherosclerosis progression, oxidized lipoproteins accumulate in macrophages and other subendothelial cells of the vascular wall provoking an inflammatory response and the progression of the plaque formation. The contribution of circulating (not local) MV in atherothrombosis needs further research.

**Figure 2:**
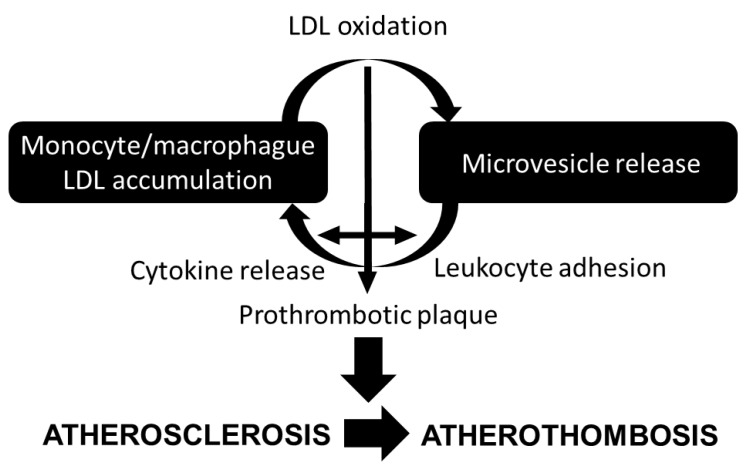
Lipid and microvesicle crosstalk contributing to atherosclerosis progression.

As previously mentioned and summarized in Figure 2, monocyte-derived macrophages accumulate cholesterol modified within the arterial wall during atherogenesis [54]. In its turn, cholesterol accumulation in macrophages and foam cells enhance AV^+^ and tissue factor^+^ MV release [55], potentially contributing to the prothrombotic state in hypercholesterolemia [56], and to the prothrombotic core of the lipid-rich vulnerable plaque [57]. 

It is known that oxidized low-density lipoprotein (oxLDL) activates platelets [58,59,60], thus inducing a prothrombotic state [39]. In addition, oxLDL induces the cellular release of MV [33,39]. In fact, total, AV^+^ and CD41a^+^ MV release was shown increased after challenging platelets with oxLDL (but not native LDL) [61], and this effect was comparable to that of ADP. However, Nielsen et al. [62] observed that *in vitro* incubation of platelets with oxLDL (or native LDL as well) did not significantly stimulate CD41^+^ and CD41^+^/CD36^+^ MV release, suggesting that MV release induced by oxLDL may not be mediated by the interaction with CD36 on platelets. 

It has been shown that oxLDL, in a time- and dose-dependent manner, promotes the *in vitro* release of endothelial MV rich in intercellular adhesion molecule 1 (ICAM-1) [63], which can be transferred from MV to endothelial cells increasing monocyte adhesion to endothelial cells [64], further propagating the atheroprone effects of oxLDL even in its absence. Moreover, enrichment of THP-1 monocytic cells with unsterilized cholesterol resulted in increased MV production [65], and these MV induced extensive leukocyte rolling and adherence to the endothelium. In the presence of oxLDL, high shear stress-induced platelet-derived MV were able to activate THP-1 monocytes and induce them to generate tissue factor-rich MV in vitro [66]. 

Elevated concentrations of circulating autoantibodies for oxLDL, a surrogate biomarker for LDL oxidation in vivo, have been associated with increased levels of platelet- and monocyte-derived circulating MV in acute coronary syndrome patients [66]. As previously stated, oxLDL induces the cellular release of tissue factor-exposing MV, promoting coagulation and thrombosis, and also disseminating the inflammatory response [39]. Although surface molecules of parental cells are transferred to MV, the interactions between of oxLDL and MV are not entirely elucidated and the consequent effects still remain unknown.

In addition to oxidized LDL, aggregated LDL but not native LDL increases tissue factor-loaded MV from smooth muscle cells [67]. In their turn, oxidized MV have been shown to stimulate monocyte adhesion to endothelial cells through oxidized membrane phospholipids, thus also contributing to atherosclerosis progression [65,68,69].

Given the heterogenic composition of MV, they may have cardioprotective functions as well. As recently reviewed, some MV carry antioxidant enzymes, conferring antioxidant activity at MV under specific stimuli [70]. In its turn, MV have also been shown to both stimulate or inhibit angiogenesis by several mechanisms of action, again depending on the cellular origin and molecular composition derived from the trigger or condition originating their release [71].

## 5. Role of Microvesicles in Dyslipidemia

The pathophysiological link between postprandial hypertriglyceridemia, inflammation and endothelial injury may be provoked by an excessive retention of lipoproteins in the extracellular matrix and increased uptake by macrophages, thus initiating the atherogenic process. A large body of evidence indicates a direct relationship between postprandial hypertriglyceridemia and CVD risk [72,73]. Postprandial dyslipidemia, independently of the caloric intake or the postprandial state itself, is associated with increased endothelial-derived CD31^+^/CD42^−^ [74], and total circulating MV levels in healthy subjects [75], and to increased platelet-derived MV in men with different CV risk burden [76], in parallel to increased markers of oxidative stress such as oxLDL and impaired flow-mediated dilation [75]. The effects of postprandial hypertriglyceridemia on MV release in subjects with metabolic dysregulation are quite unexplored and deserve further research, as the reported results are controversial. Type 2 diabetic patients, who show exacerbated postprandial dyslipidemia, show around 3.5 fold increased concentration of endothelial-derived CD144 circulating MV after a meal [77]. However, patients with carotid atherosclerosis show a similar postprandial elevation of circulating platelet-derived MV, despite having higher postprandial hypertriglyceridemia than control subjects free of atherosclerosis [78].

Patients with hypercholesterolemia show higher levels of monocyte- and platelet-derived MV than healthy subjects [79,80,81]. Familial hypercholesterolemia (FH) is an autosomal dominant genetic disorder associated with elevated LDL cholesterol levels and deposition in tendons (xanthomas), and premature heart disease [82]. MV have been associated with atherosclerosis progression and with a higher risk of atherothrombosis in FH patients. In fact, elevated concentrations of total, endothelial cell-derived, erythrocyte-derived, monocyte-derived, tissue factor-loaded MV [83], and platelet-derived MV [84], have been found in FH patients compared to healthy controls [85], and identify subclinical atherosclerosis [80]. In addition, circulating CD36^+^ MV derived from endothelial cells and monocytes were significantly higher in FH patients compared to healthy controls [85], and monocyte-derived circulating MV in FH patients directly correlated with oxLDL plasma concentrations [79]. Moreover, these patients showed increased concentrations of circulating MV derived from leukocytes, and lymphocyte-derived CD3^+^/CD45^+^ circulating MV have been shown elevated in FH patients with lipid-rich atherosclerotic plaques [86]. In addition, platelet- (CD41a^+^/AV^+^, CD31^+^/AV^+^, CD41a^+^/CD31^+^/AV^+^), granulocyte- (CD66^+^/AV^+^), neutrophil- (CD11b^+^/CD66^+^/AV^+^), and endothelial cell-derived (CD62E^+^/AV^+/−^) circulating MV discriminate and map coronary atherosclerotic plaque and calcification [84], and leukocyte- and activated platelet-derived circulating MV predict a major cardiovascular event in these patients three years before it takes place [87]. 

In FH patients, plasma apheresis reduces circulating MV concentration, mainly platelet-derived MV AV^+^ [88].

### Pharmacological Treatment of Dyslipidemia and Circulating MV

Given the crosstalk between lipids and MV, as discussed in a previous section, it appears plausible that lipid lowering therapy may elicit a pleiotropic effect by (partially) inhibiting MV release, contributing to explain their beneficial effects in atherothrombotic complications. We have observed that statins reduce MV shedding from platelets, endothelial cells and leukocytes carrying markers of cell activation [89], and this effect was found to be accumulative throughout years of treatment. Other researchers have also observed that statins decrease MV release from several cell origins [90,91,92], and modify their molecular fingerprint by decreasing their cargo of cell activation markers [93,94]. In stroke patients with hyperlipidemia, simvastatin treatment for 6 months reduced the percentage of CD61^+^ platelet-derived MV to similar levels to those of age- and sex-matched controls [95].

However, some authors did not observe such effect of statins [96,97], and, as discussed in the 2019 ESC/EAS Guidelines for the management of dyslipidemias, the clinical relevance of the pleiotropic effects of statins remains clinically unproven [98], and thus may be object of future research.

## 6. Conclusions and Future Perspectives

The studies summarized in this review evidence that MV are settled in the tandem lipids inflammation, contributing to the progression of atherothrombosis leading to a major CV event. Despite a huge body of research towards the understanding of the exact contribution of MV in this process, several questions remain unanswered, such as the specific interaction of MV with native and modified LDL, the molecular fingerprint of MV, how do they qualitatively (by which pathways) and quantitatively (in what amounts) contribute in the presentation of major CV events, and if they can be considered real candidates as drug delivery vectors in the clinical setting. Given the central role of MV in systemic inflammation and endothelial injury within the pathophysiology of atherothrombosis, their link with lipids and lipoproteins are of particular interest. Understanding the pathways implicated in this process will aid in developing novel therapeutic approaches against early atherosclerosis. In this setting, MV and lipoproteins exhibit common features, which should be considered in the interpretation of their respective roles in the pathophysiology of CVD.
